# Management of Hemodynamically Unstable Pelvic Ring Fractures

**DOI:** 10.3389/fsurg.2020.601321

**Published:** 2020-12-04

**Authors:** Kim E. M. Benders, Luke P. H. Leenen

**Affiliations:** Department of Surgery, University Medical Center Utrecht, Utrecht, Netherlands

**Keywords:** pelvic fracture, hemodynamically unstable, pelvic packing, external fixation, angioembolization

## Abstract

Hemodynamically unstable pelvic fractures are challenging high-energy traumas. In many cases, these severely injured patients have additional traumatic injuries that also require a trauma surgeon's attention. However, these patients are often in extremis and require a multidisciplinary approach that needs to be set up in minutes. This calls for an evidence-based treatment algorithm. We think that the treatment of hemodynamically unstable pelvic fractures should primarily involve thorough resuscitation, mechanical stabilization, and preperitoneal pelvic packing. Angioembolization should be considered in patients that remain hemodynamically unstable. However, it should be used as an adjunct, rather than a primary means to achieve hemodynamic stability as most of the exsanguinating bleeding sources in pelvic trauma are of venous origin. Time is of the essence in these patients and should therefore be used appropriately. Hence, the hemodynamic status and physiology should be the driving force behind each decision-making step within the algorithm.

## Introduction

Pelvic ring injuries with concomitant hemodynamic instability is one of the most challenging high-energy traumas. It often involves young polytrauma patients that have been in motor vehicle accidents, pedestrian collisions or fall from heights. The incidence of life-threatening pelvic trauma is only 1–4% of all pelvic injuries (1), making it a relatively rare condition requiring specialized care in a race against rapid on-going exsanguination. The management of such injuries necessitates a multidisciplinary approach, involving multiple medical specialties such as emergent care physicians, trauma surgeons, orthopedic surgeons, and intervention radiologists. Mortality rates are as high as 30–60% and are related to exsanguination and associated neurological, thoracic and/or abdominal injuries (2–5).

Appropriate decision making in treating these severely injured patients starts by identifying those in need of urgent care. Pelvic fractures are generally classified according to the Young and Burgess (6) classification which discriminates according to the mechanism of injury, such as lateral compression, anterior-posterior compression and vertical shear injuries. Especially severe anterior-posterior compression injuries, such as open book injuries, and vertical shear injuries are frequently associated with vascular compromise. However, the anatomical description of the Young and Burgess classification does not aid in determining the prognosis of a pelvic fracture patient. To this extent the World Society of Emergency Surgery (WSES) classification has been proposed and validated (7). This classification stratifies patients according to vascular injury severity in combination with the mechanical instability of the pelvic ring. As expected, the highest grade of injury (WSES grade IV) involves mechanically unstable pelvic ring injuries and hemodynamically unstable patients which require aggressive resuscitation and interventions to control the ongoing hemorrhage.

In general, the treatment of severe pelvic trauma can involve resuscitation, mechanical stabilization, pelvic packing, and angioembolization. However, the order in which these modalities are used seems to be largely geographically dependent. The European approach (8–11) tends to focus more on pre-peritoneal pelvic packing, whereas the American approach tends to lean more toward angioembolization (5, 12, 13). However, in the past few years, preperitoneal pelvic packing has also gained ground within American practice (2, 14). Nonetheless no clear guidelines exist and no consensus has been reached in how to approach hemodynamically unstable pelvic fractures. This will lead to variable approaches and consistently high mortality rates.

**Figure 1:**
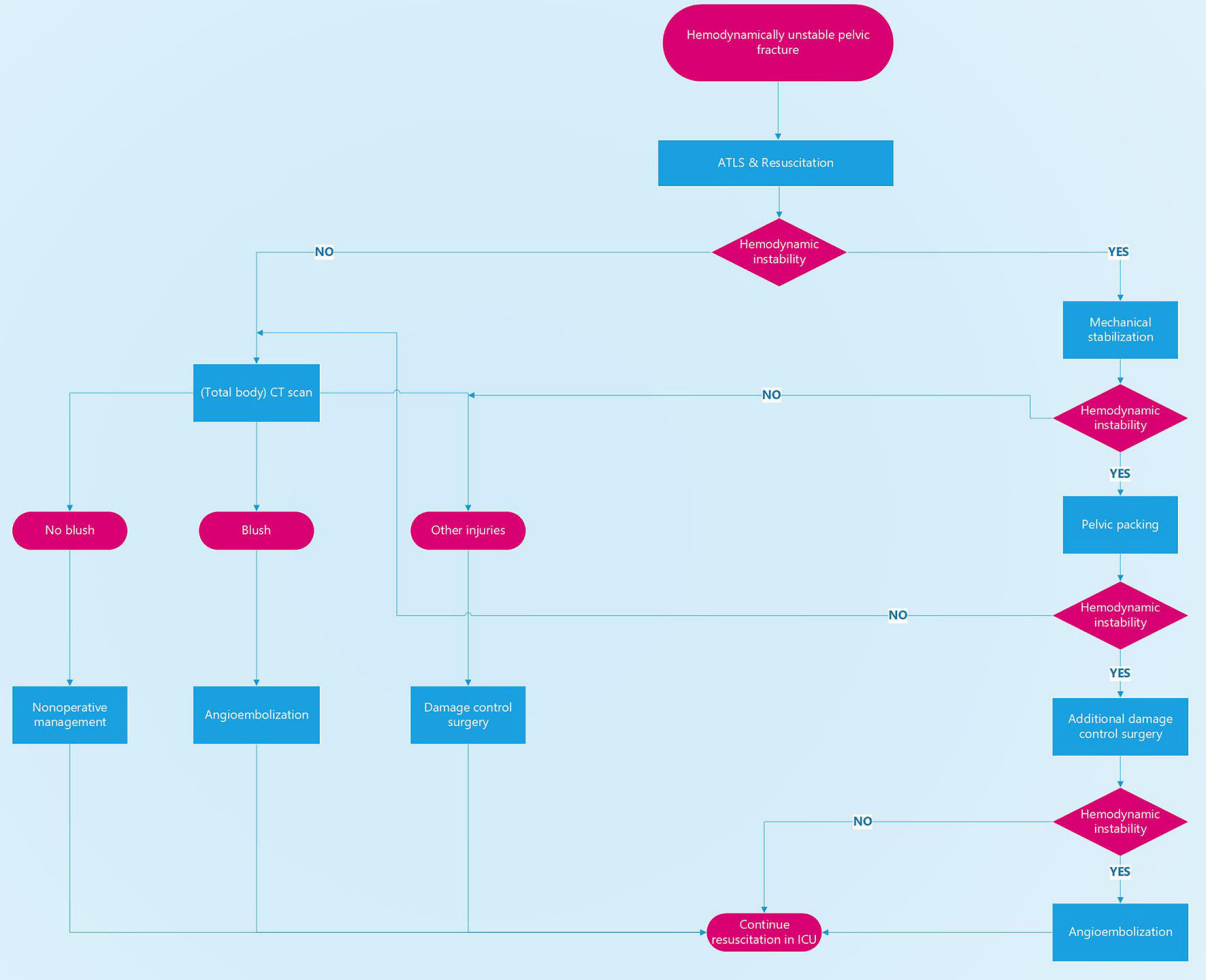
Treatment algorithm for hemodynamically unstable pelvic fractures. Decision-making is based on the patient's physiological status. Hemodynamically unstable patients should not be taken to a CT scanner or to the angio suite but to the operating theater for mechanical stabilization, pelvic packing, and additional damage control surgery if needed.

We think that early control of hemorrhage is paramount to increasing survival numbers. We therefore think that the patient's physiological response to resuscitative measures should be the lead discriminator in deciding the sequence of the possible treatment modalities (Figure 1).

### Primary Care of the Pelvic Trauma Patient and Resuscitation

Severe pelvic trauma rarely presents itself as a mono-injury. High incidences up to 80% of associated neurological, thoracic, and intra-abdominal injuries have been reported due to the extreme energy forces that are transferred through the body when sustaining severe pelvic trauma (2, 15–17). All polytrauma patients require a standardized diagnostic work-up strategy in the emergency room to prevent unnecessary and potentially fatal delay in treatment. Primary evaluation according to the guidelines of Advanced Trauma Life Support (ATLS) has been adopted worldwide and provides a rapid sequence in which most life-threatening conditions can be identified and treated. The hemodynamically unstable patient has further been defined according to ATLS guidelines with blood pressure <90 mmHg, heart rate >120 bpm, altered consciousness and/or shortness of breath. By using a clear definition of hemodynamic instability like this, or any other shock index, all physicians will have a clear idea of the severity of the injuries as well as the need for prompt interventions after locating the origin of the blood loss according to the motto “blood on the floor and four places more”.

One of the cornerstones in the treatment of hemodynamically unstable patients is the early activation of massive transfusion protocols (18). All hemodynamically unstable patients should be considered eligible to receive transfusion of red blood cells, plasma, and thrombocytes in a 1:1:1 ratio to halt massive blood loss and to prevent further metabolic deterioration. In case patients are on anti-thrombotic therapy, swift administration of coagulating agents should be considered. In case of pelvic trauma, the use of massive transfusion has shown to reduce the need for further surgical intervention (8). Whenever a patient seems to be responder (return to normal vital signs), or even a transient responder (transient improvement, however recurrence of hemodynamic instability) to resuscitative measures in the emergency room further diagnostic procedures can be initiated (Figure 1). In case of persistent hemodynamic instability in the trauma bay in a pelvic trauma patient with a mechanically unstable pelvis it is of utmost importance to stabilize the pelvis and allow for local hemostasis.

### Mechanical Stabilization

**Figure 2:**
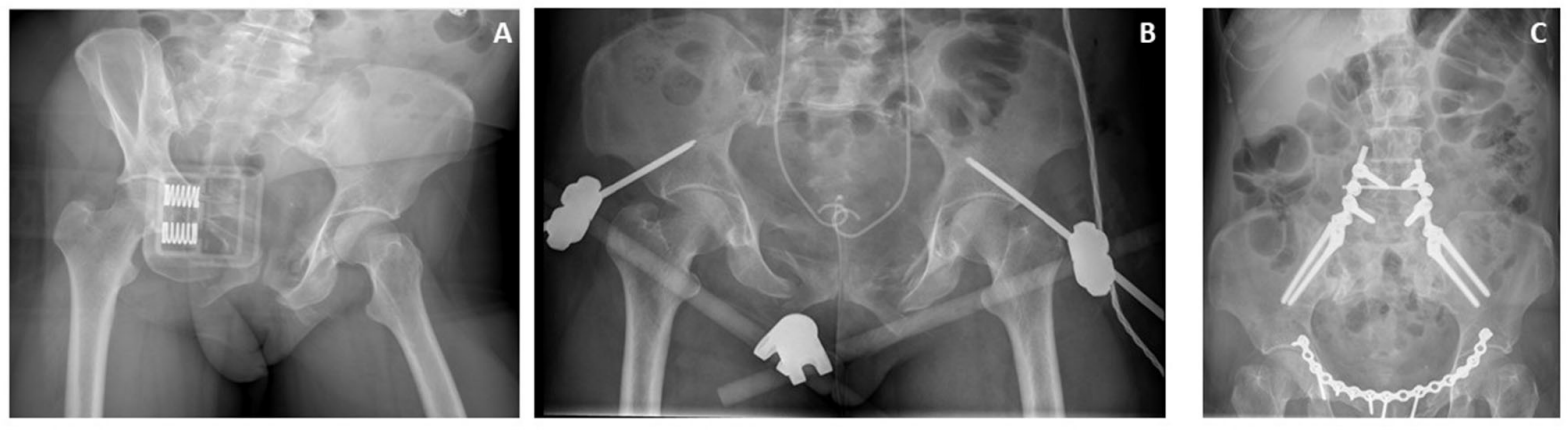
Case of a patient with a hemodynamically unstable pelvic fracture. **(A)** closure of the pelvic space using a pelvic binder. **(B)** Supra-acetabular external fixator, and JJ stents after first surgical intervention. **(C)** Definitive repair including pelvic ring plating, and lumbosacral fixation.

Pelvic fracture stabilization can be achieved quickly in the emergency room or even in the pre-hospital setting by using a pelvic binder of any kind. They are recommended as a primary strategy in applying compression and closing the pelvic ring in the early treatment of hemorrhagic instability when positioned correctly (around the greater trochanters) in combination with adduction of the feet. Adduction of the feet can, however, not be achieved when femoral fractures are present. Their main advantages are that they are non-invasive, only take a few minutes to apply, and do not require any surgical training. A pelvic binder does not only immobilize the pelvis allowing for clot formation in a stable hematoma, but it also greatly diminishes pelvic volume, especially in AP compression injuries (Figure 2). Pelvic binders have been shown to reduce the volume with ~10%, which is equal to the volume reduction that can be obtained when placing an external fixator (19). Pelvic dislocation of only 3 cm can already lead to an increase in volume of 1.5 L, which can strongly attribute to rapid exsanguination of a trauma patient. Early application of a pelvic binder has been shown to reduce the amount of blood products that were required to acquire hemodynamic stability (20). However, overall mortality was not brought down its early application.

**Figure 3:**
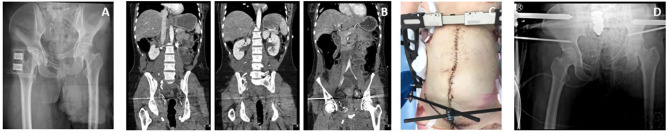
Case of a patient in which a pelvic binder was used in the ED to temporarily stabilize the pelvis **(A)**. After initial surgical stabilization with a supracetabular external fixator and pelvic packing a CT scan was made showing additional injury to the posterior side of the pelvic ring **(B)**. Because of persisting posterior mechanical instability a C-clamp was also used to further reduce the pelvis **(C,D)** and allow for better control of the pelvic bleeding.

Despite the swiftness with which a pelvic binder can be positioned, it is not a treatment modality that can be left on indefinitely. Therefore, this binder is often replaced by external fixators or C-clamps either in the emergency room or in the operating theater (Figures 2, 3). Anterior external fixation is more commonly used to stabilize the pelvis than the C-clamp (1). It is an orthopedic procedure that does not require extensive training and allows for appropriate reduction of especially anterior-posterior compression injuries. The Schanz pins can be introduced in the iliac crest or supra-acetabular. Iliac crest insertion is less technically demanding, however the pull-out strength is much lower and may lead to early loss of reduction and fixation leading to insufficient hemorrhage control. Supra-acetabular placement of the Schanz screws leads to stronger fixation and reduction but does often require C-arm guidance to ensure appropriate placement meaning that this procedure takes up slightly more time.

Vertical shear injuries lead to a multi-directional unstable pelvis. This type of injury requires posterior stabilization, which cannot be achieved using an anteriorly placed external fixator. Moreover, strong anterior reduction may lead to opening up of the posterior pelvis leading to an increase in pelvic volume and subsequent hemorrhaging. The preferred method of external fixation in vertical shear injury patterns is the C-clamp (Figure 3). It has been shown to be a reliable external fixator for posterior pelvic injuries when taking into account the anatomical landmarks (21). Unfortunately, this procedure is not frequently used worldwide due to the relatively low incidence of hemodynamically unstable vertical shear injuries, leading to a loss of expertise. Contra-indications for the placement of a C-clamp are severely comminuted sacral fractures, certain iliac wing fractures (crescent fracture) or lateral compression injuries. Its main complications are nerve damage and pin perforation (20, 21).

Some trauma surgeons advocate the use of an antishock iliosacral screw, or rescue screw (22). This involves the placement of a cannulated iliosacral screw instead of posterior stabilization with a C-clamp. However, in our current practice not all trauma surgeons in our institution are pelvic surgeons and are therefore less inclined to perform antishock iliosacral screws. Moreover, a prerequisite to the insertion of antishock iliosacral screws is state of the art intraoperative imaging, that may not be present in all institutions around the world caring for these patients.

The motivation for using external fixation in hemodynamically unstable pelvic fractures is two-fold. First of all, it reduces the pelvic space, leading to volume reduction (20), and it aligns the bony fragments from which persistent bleeding may occur. Secondly, it aids in offering counter pressure against the pre-peritoneal lap sponges that are used in pre-peritoneal packing which is the next step in treating hemorrhaging pelvic fractures. The appropriate application of an external fixator and especially a C-clamp will not intervene with the surgical field and should be applied prior to any preperitoneal or intra-abdominal exploration.

### Preperitoneal Packing

Up to 80% of all bleeding from the pelvis is from a venous origin. The most injured veins are the presacral plexus, and the prevesical veins. In only 20% of all pelvic injuries will there be an arterial laceration leading to blood loss. In these cases, the arteries that are mostly involved are the branches of the internal iliac artery, pudendal artery, obturator artery, superior gluteal artery and lateral sacral artery. Next to the possible vascular disruptions, a discontinuation of pelvic bones is a third source of hemorrhage in pelvic trauma.

In general, arterial injuries tend to respond well to angioembolization, venous bleeds are notorious to be inadequately managed by angioembolization. As only 20% of all bleeding in hemodynamically unstable pelvic fractures has an arterial origin, it seems rather counterintuitive to focus treatment on arterial injury when there is an overwhelming 80% of venous injuries that will continue to hemorrhage whilst performing angioembolization. Also in case of arterial bleeding, there is a 100% chance of concomitant venous bleeding, again warranting the necessity to address the venous hemorrhage. For this reason pre-peritoneal packing has gained interest as a primary means to gain hemorrhage control after mechanical stabilization of the pelvis (11). Pre-peritoneal packing has been shown to be an effective means to control low-pressure hemorrhage from venous sources as well as from blood extravasating from the bone (1). It is a quick, and relatively easy surgical procedure as the approach to the pre-peritoneal space is clear cut and the dissection has often already been achieved by the hemorrhage (11, 14, 23, 24). Local compression using surgical lap pads allows for hemostasis to occur in the confined space of the pelvis that has previously been stabilized with a external fixator or a C-clamp.

Retrospective studies have shown that implementing preperitoneal packing as a primary treatment strategy in hemodynamically unstable pelvic fractures after external fixation leads to a reduction of ~12% in hemorrhage related fatalities (24, 25).

Despite the pragmatic approach in dealing with hemorrhage at its most likely origin, we believe pre-peritoneal packing to be of great value due to speed in which a patient can be prepped (Figure 2). In hemodynamically unstable patients, every second counts and none should be wasted to gain control of the bleeding. In most level one trauma centers, angio suites are available 24/7. However, prepping the angio suite and getting all required personnel into the hospital is more challenging and takes more time than having a trauma surgeon on call and ready to perform surgery. Burlew et al. (14) has shown that not only the time until the start of the procedure was significantly shorter in pelvic packing (55–79 min) than in angioembolization (140–194 min), but also the procedural time is a lot shorter in pre-peritoneal pelvic packing in combination with external fixation (44 min) than angioembolization alone (193–301 min). During the time that elapses in preparing angio suites the patient generally remains hemodynamically compromised, thereby increasing the risk of sudden deterioration and death. We believe that this should be prevented by all means and therefore we do not advocate angioembolization in hemodynamically unstable patients. Moreover, as angioembolization is a time-consuming procedure lives may be lost due to exsanguination during the procedure (23), suggesting that pre-peritoneal pelvic packing is a safer option in hemodynamically compromised patients. Also, when comparing pre-peritoneal pelvic packing and angioembolization as a primary treatment option, patients required less transfusions in the first 24 h after treatment when they were treated with pre-peritoneal packing (4). As increased demand for transfusion is related with higher mortality rates, we can deduce that pre-peritoneal packing will have a positive effect on reducing mortality numbers.

As hemodynamically unstable pelvic trauma patients often suffer from additional injuries which may be a source of bleeding, these should also be addressed after control of the pelvic hemorrhage. In these cases crash laparotomy may be required to explore the abdominal cavity. It should be noted however, that without prior mechanical stabilization of the pelvis by either a pelvic binder, external fixation or C-clamp, a crash laparotomy will lead to immediate loss of local tamponade and an increased bleeding incentive (10). A 100% mortality rate has been reported in patients undergoing laparotomy or angiography without prior external fixation (10). However, if both pre-peritoneal packing has been performed and other sources of blood loss have been controlled and the patient remains hemodynamically compromised and continuously requires blood or plasma transfusions, angioembolization should be considered to rule out a arterial bleeding source and to treat it accordingly.

### Angioembolization

Angioembolization should be used as a complimentary treatment modality in which further hemodynamic stability can be acquired after external fixation of the pelvic ring, adequate preperitoneal packing in combination with ample resuscitation has been performed. Cothren et al. (2) have shown that only 13% of all patients require secondary angioembolization in addition to external fixation and packing. This coincides with the up to 20% arterial injuries that are generally found in hemodynamically unstable pelvic fractures. Also, exclusive use of angioembolization without pre-peritoneal packing or applying mechanical stabilization has shown to lead to higher mortality in patients with severe pelvic trauma (26).

One of the most predictive signs in the need for angioembolization is the presence of an arterial blush on CT imaging. However, in our institution we do not take hemodynamically unstable patients to the CT scan, instead we perform damage control surgery by temporary mechanical stabilization and preperitoneal packing. After initial stabilization in the operating theater, there should be a window of opportunity to perform a dual-modality CT scan to evaluate other hemorrhage sources such as for example persistent arterial bleeds within the pelvic area. Only then do we consider angioembolization of the attained arterial blush.

**Figure 4:**
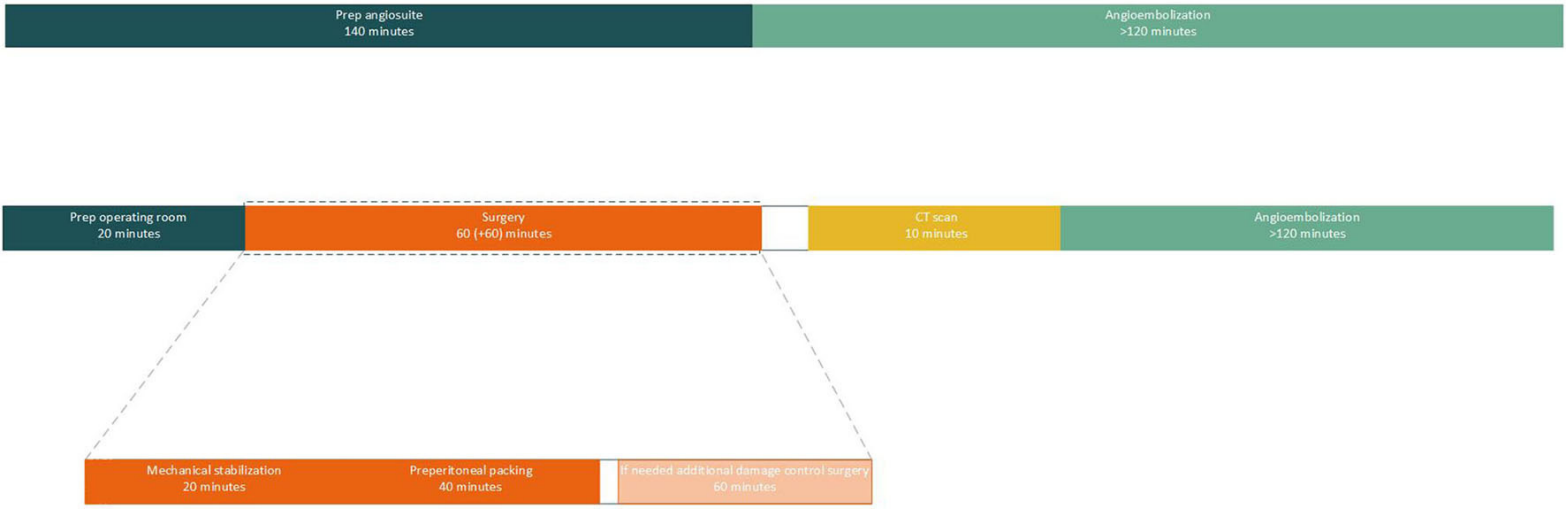
An overview of the time line of both angioembolization and mechanical stabilization, pelvic packing, CT scan and additional angioembolization. Procedural times and prepping times are extracted from real life cases and extracted from Burlew et al. (14). This overview shows that the time needed for prepping an operating theater and performing mechanical stabilization, preperitoneal packing and if needed additional damage control surgery, can be used to prepare the angio suite for additional angioembolization if required. It also becomes evident that preparing an angio suite causes an unnecessary delay in treatment in hemodynamically unstable patients.

As mentioned before angioembolization is a time-consuming process and should therefore not be the first line of defense in exsanguinating pelvic trauma (23). However, in severe pelvic trauma in which both venous and arterial damage is suspected, the time spent in the operating theater for mechanical stabilization and pelvic packing can be well-spent on preparing the angio suite for secondary angioembolization (Figure 4). Moreover, the chance of patients exsanguinating during angioembolization will be diminished as venous bleeding sources will have already been addressed in the operating theater.

### REBOA

Resuscitative endovascular balloon occlusion of the aorta (REBOA) involves a minimally invasive procedure that can be considered in damage control in case of exsanguinating hemorrhage (27). It was first used in major exsanguinating warfare injuries, but its use is slowly been adopted to civilian trauma cases in which major hemorrhage occurs. By occluding the aorta with an endovascular balloon that can be introduced through the femoral artery, temporary hemorrhage control may be obtained. The use of resuscitative endovascular balloon occlusion of the aorta (REBOA) has however not been widely adopted in the treatment of hemodynamically unstable pelvic fractures. REBOA is relatively new and therefore still has extensive learning curves and should only be performed by trained acute care surgeons or interventionalists. In exsanguinating patients, inserting the balloon catheter is even more difficult as blood pressure drops. Complications of REBOA can be severe and bring the resuscitative effort to a premature end due to arterial disruption or dissection or lead to future problems due to thromboembolism or limb/organ ischemia (28, 29). However, REBOA can be considered by trained physicians in patients in extremis. In the hands of trained physicians it can be seen as an equivalent to a resuscitative thoracotomy for aortic cross-clamping in critically uncontrollable hemorrhagic shock.

When comparing preperitoneal packing to the use of REBOA in hemodynamically unstable pelvic fractures, the latter seems to be inferior to packing (30, 31). Despite the more rapid initiation of treatment in the REBOA group compared to the packing group, the time spent in the emergency department was longer, and in-hospital mortality was higher after REBOA (30). This again seems to underscore the need to treat venous hemorrhage prior to addressing arterial pelvic bleeding.

At most REBOA should be used as a bridge to other treatment modalities. However, when using REBOA to bridge to angioembolization, mortality rates are as high as 46% (32). In our opinion these are unacceptable high fatality rates and refinement of the technique is required prior to adapting this to the algorithm for the treatment of hemodynamically unstable pelvic fractures.

## Conclusion

Hemodynamically unstable pelvic fractures are life-threatening injuries that require a multidisciplinary approach and standardized care in order to bring down the high mortality rates. In order to achieve this, treatment algorithms should be implemented and the care for such patients should be centralized. In the United States, 25% of all unstable pelvic fractures are first presented to small hospitals which may not be equipped to treat these potentially life threatening injuries (33). Admittance to level one trauma centers with appropriate on call specialists will give patients the best chance of survival if clear treatment guidelines are in place.

We advocate that early mechanical stabilization in combination with preperitoneal packing as the optimal, evidence-based approach for the timely treatment of hemodynamically unstable pelvic fractures. Angioembolization should be considered as an optimal adjunct whenever a patient remains in need of transfusion when all other bleeding sources have been controlled.

## Author Contributions

The manuscript was drafted by KB and LL. Critical review was performed by LL. Both authors contributed to the article and approved the submitted version.

## Conflict of Interest

The authors declare that the research was conducted in the absence of any commercial or financial relationships that could be construed as a potential conflict of interest.
